# Preparation of Diamond Nanofluids and Study of Lubrication Properties

**DOI:** 10.3390/ma18092052

**Published:** 2025-04-30

**Authors:** Jiamin Yu, Junhao Wu, Chengcheng Jiao, Huanyi Chen, Xinxin Ruan, Wei Li, Genxiang Gong, Jinhong Yu, Kazuhito Nishimura, Nan Jiang, Tao Cai, Zhisheng Wu

**Affiliations:** 1 Department of Materials and Chemical Engineering, Taiyuan University of Science and Technology, Taiyuan 030024, China; yujiamin@nimte.ac.cn; 2 State Key Laboratory of Advanced Marine Materials, Ningbo Institute of Materials Technology and Engineering, Chinese Academy of Sciences, Ningbo 315201, China; wujunhao@nimte.ac.cn (J.W.); jiaochengcheng@nimte.ac.cn (C.J.); chenhuanyi@nimte.ac.cn (H.C.); ruanxinxin@nimte.ac.cn (X.R.); liwei22@nimte.ac.cn (W.L.); gonggenxiang@nimte.ac.cn (G.G.); yujinhong@nimte.ac.cn (J.Y.); kazuhito@kcb-net.ne.jp (K.N.); jiangnan@nimte.ac.cn (N.J.); 3 Advanced Nano-Processing Engineering Laboratory, Mechanical Engineering, Kogakuin University, Tokyo 192-0015, Japan

**Keywords:** 2D diamond nanosheets, nanofluid, lubricant additives

## Abstract

As an emerging two-dimensional nanomaterial, diamond nanosheets have the advantages of high hardness and chemical stability; exhibiting good tribological properties when used as lubricant additives. However, the dispersion stability of nanomaterials as additives in lubricants remains a significant challenge. In this study, fluidized and functionalized diamond nanofluids were prepared by grafting polyether amine on the surface of diamond nanosheets. By changing the state of diamond nanosheets, this material not only improved its own lubrication property, but also improved its dispersion in the lubricant. The friction test results demonstrated that the friction coefficient was reduced by 66.9% and the wear rate was reduced by 81.8% with the addition of 3 wt% of diamond nanofluid in water–glycol solution. This enhancement of lubricating properties is related to the excellent film-forming properties of diamond nanofluids during the tribology. This **indicates** that fluidized 2D diamond nanosheets have excellent lubricating properties and can significantly improve the friction properties of lubricants as additives.

## 1. Introduction

Globally, friction and wear account for approximately 20% of the total energy consumed every year [1,2]. Current studies have shown that the use of liquid lubricants serves as an effective means of reducing friction and wear problems [3,4]. Liquid lubricants convert friction from the contact surface to the fluid interior; thus, lower-viscosity lubricants have the potential to achieve lower coefficients of friction. However, for lubricants, viscosity is directly proportional to pressure resistance, and low viscosity will lead to higher wear and failure of lubrication at load [5,6,7]. Therefore, it is difficult to achieve reduced friction and wear simultaneously with a single lubricant. Moreover, when the friction equipment is in startup, shutdown, or under extreme friction conditions, liquid lubricants are unable to form a stable lubricating film between the friction pairs, making it prone to boundary lubrication and mixed lubrication states, which can result in severe friction and wear [8,9]. The use of lubricant additives is one of the effective methods to reduce friction and wear under boundary lubrication conditions. Among the many lubricant additives, nanomaterials have garnered widespread attention from researchers due to their small size, which allows them to easily enter frictional areas and form protective lubricating films [10,11,12]. In particular, two-dimensional nanomaterials have become a key focus in the field of tribology research due to their large specific surface area, and superior physical and chemical properties [13,14].

With the initial discovery of graphene, an increasing number of 2D nanomaterials have been reported and studied in recent years; including molybdenum disulfide, hexagonal boron nitride, and MXenes [2]. These 2D nanomaterials with unique planar structures and favorable physicochemical properties have attracted much attention in lubricant additives due to their higher anti-wear properties and better lubricating ability due to interlayer low-shear interaction [15,16]. Wu et al. [17] prepared graphene oxide as a raw material to obtain sulfonated graphene with better lubricating properties, which was used as a lubricating additive for water. It was demonstrated in the study that the addition of 0.2 wt% sulfonated graphene resulted in a 25.8% increase in water viscosity, a 74% reduction in coefficient of friction (COF), and a 15.7% reduction in wear. Cui et al. [18] prepared an MXene@CD hybrid from carbon dots and MXene, which was used as an additive for a PAO lubricant. The experimental results demonstrated that the COF of PAO decreased from 0.58 to 0.1, and the wear decreased by 91.3% with the addition of the MXene@CD lubricant additive. Although current research demonstrated that 2D nanomaterials possessed good tribological properties when used as lubricant additives, the poor dispersion stability of these nanomaterials in lubricants remains a key issue that limits their further development. In addition, most 2D nanomaterials can be generally characterized by low mechanical strength, making it difficult to resist larger pressures and causing greater wear during friction [19]. Compared with other two-dimensional nanomaterials, the two-dimensional diamond nanosheets obtained by the latest cleavage plane crushing method have been demonstrated to have higher hardness and good chemical stability, which can effectively reduce wear during the friction process [3,20]. Moreover, the fluidization modification method of nanomaterials has been proven to effectively enhance the dispersibility and stability of nanomaterials in lubricating oils [21,22]. For example, Guo et al. [23] prepared various types of solvent-free ionic silica nanofluids, which significantly improved the dispersion of silica in PEG 400, resulting in high-performance lubrication behavior.

To address the abovementioned problems encountered in the field of 2D nanomaterials in lubrication additives, in this study, 2D diamond nanosheets, organosilane γ-glycidyl ether oxypropyltrimethoxysilane (KH560) and polyether amine (M2070), were used as the raw materials. A two-dimensional diamond nanofluid was prepared through fluidization modification. The modification method of fluidization could change the state of the diamond nanosheets; not only addressing the poor dispersion stability issue of nanomaterials in lubricants but also improving their lubrication performance. In addition, diamond nanofluids exhibit good film-forming properties in the friction process as lubricating additives, effectively reducing friction and wear.

## 2. Experimental Section

### 2.1. Materials

Diamond nanosheets (SCND, average diameters of 70/120/185 nm) were purchased from Henan Namei New Material Co., Ltd. (Zhengzhou, China) Organosilane γ-glycidoxypropyltrimethoxysilane (KH560, AR, 97%) was supplied by Aladdin Biochemical Technology Co., Ltd. (Shanghai, China) while M2070 (AR, 99%) polyether amine was obtained from Dalian Liansheng Trading (Dalian, China), and anhydrous ethanol was purchased from Sinopharm Chemical Reagent Co., Ltd. (Shanghai, China) All reagents were used as received, without further treatment.

### 2.2. Preparation of the Solvent-Free 2D Diamond Nanofluid

**Figure 1:**
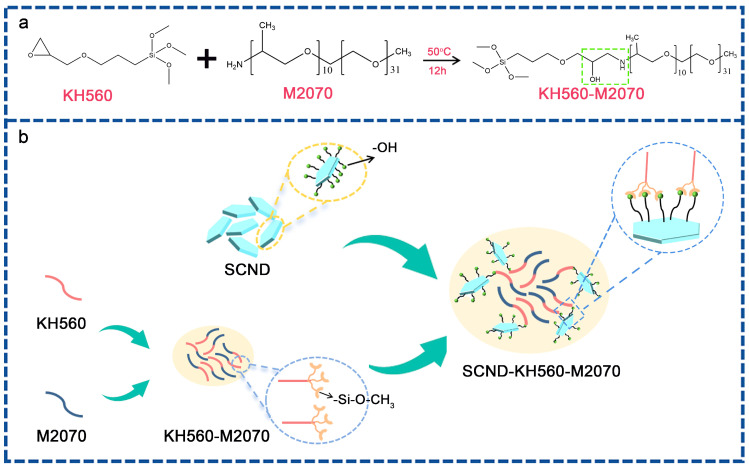
Synthesis of KH560-M2070 intermediate (**a**) and schematic diagram of the preparation process of solvent-free 2D diamond nanofluid (**b**).

In this experiment, a solvent-free, two-dimensional diamond nanofluid was prepared using KH560, M2070, and two-dimensional diamond nanosheets as raw materials. KH560 and M2070 can form an organic shell layer through covalent bonding, and the resulting product undergoes a condensation reaction with the diamond nanosheets to form the nanofluid. Figure 1a shows the reaction mechanism between KH560 and M2070. The preparation process of the solvent-free, two-dimensional diamond nanofluid is shown in Figure 1b. First, an ethanol solution containing 5 wt% KH560 is slowly added to an ethanol solution containing 10 wt% polyetheramine M2070. Then, the mixed solution is continuously stirred at 50 °C for 12 h to form covalent bonds within the KH560-M2070 organic shell layer. Next, 0.5 g of diamond nanosheets, pre-dispersed in ethanol, are mixed into the KH560-M2070 ethanol solution, and a grafting reaction is carried out at 45 °C for 6 h. Subsequently, the mixture is purified for 3 days, and excess organic shell layers are removed through dialysis, followed by solvent removal via rotary evaporation. The mixture is purified for 3 days using a dialysis membrane with a molecular weight cutoff (MWCO) of 8 kDa (Model: MD44-8000, Viskase, IL, USA) to remove polyether amine (M2070) that did not undergo chemical reactions with the surface of the diamond nanosheets. This was followed by solvent removal via rotary evaporation. The resulting sample is vacuum-dried at 60 °C for 72 h to obtain the solvent-free, two-dimensional diamond nanofluid, which is labeled as SCND-KH560-M2070 (SKM).

### 2.3. Materials Characterization

The morphology of the samples was observed by a scanning electron microscope (SEM, Zeiss, Jena, Germany, G300) and a transmission electron microscope (TEM, Thermo Fisher Scientific Waltham, MA, USA, Talos F200X) equipped with an energy dispersive spectrometer (EDS). Fourier transform infrared spectrometry (FTIR, Thermo Fisher Scientific IS50) and Raman spectrometry (Raman, Horiba, Kyoto, Japan, LabRAM HR Evolution) were also used to analyze surface groups and chemical structures. The phase structures of the samples were studied by X-ray diffraction (XRD, Bruker, Billerica, MA, USA, ADVANCE D8, Cu Kα source, λ = 1.54 Å) [24], and the chemical compositions of SCND and SCND-KH560-M2070 were analyzed by X-ray photoelectron spectroscopy (XPS, Axis Ultra DLD Kratos Analytical, Manchester, UK). Thermogravimetric analysis (TGA, Netzsch, Selb, Germany, TGA 209 F1 Libra) was conducted under nitrogen protection; with the temperature increasing from 30 °C to 1000 °C (heating rate of 10 °C/min) to characterize the composition and thermal stability of the samples. Differential scanning calorimetry (DSC, TA Instruments, New Castle, DE, USA, DSC 2500) was conducted at a heating rate of 10 °C/min to investigate the thermal properties of SCND-KH560-M2070, and the rheological properties of SCND-KH560-M2070 were investigated by a rheometer (TA Instruments, Discovery HR-2).

### 2.4. Tribological Test

To evaluate the tribological properties of the diamond nanofluids with different sizes, friction tests were performed using a multifunctional high-temperature friction and wear tester (UMT, Bruker Corporation UMT-3). A ball 6.0 mm in diameter was utilized, which was composed of GCr15, and the corresponding friction partner steel plates were composed of AISI 52100 with dimensions of 2 × 2 × 1 cm^3^. The constant experimental parameters used in the friction tests—such as load, frequency, and test duration—are summarized in Table 1. Prior to the tribological test, the GCr15 steel balls and AISI 52100 steel plate were ultrasonically cleaned with petroleum ether and anhydrous ethanol. To investigate the effects of variable parameters, different sizes (70, 120, and 185 nm) and concentrations (0.5%, 1%, 3%, and 5%) of diamond nanofluids were evaluated, as detailed in Table 2. The friction tests were conducted under controlled conditions, with a stroke length of 5 mm and a sliding speed of 50 mm/s. After testing, the wear tracks were analyzed using a 3D optical profiler (Rtec Instruments, San Jose, CA, USA, UP-Lambda). To enhance the reliability of the results, three replicate measurements were performed for each sample.

## 3. Results and Discussion

### 3.1. Characterization of the Solvent-Free 2D Diamond Nanofluids

**Figure 2:**
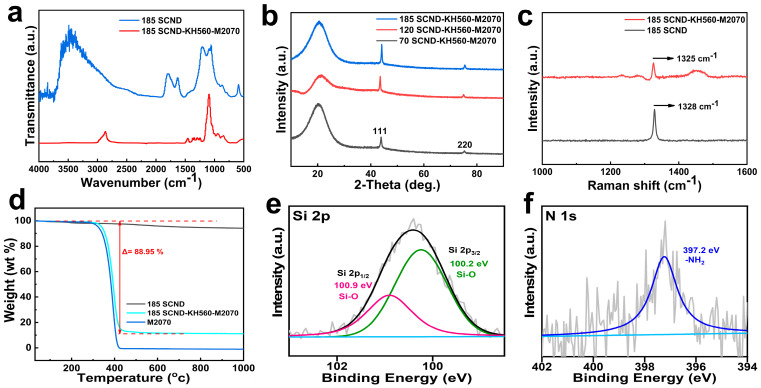
FTIR spectra (**a**), XRD of diamond nanofluid (**b**), Raman spectral analysis (**c**), and TGA (**d**) of the diamond nanofluids and corresponding solvent-free nanofluids; XPS Si 2p spectra (**e**) and N 1s spectra (**f**).

In FTIR analysis (Figure 2a, with 185 nm diamond sheet used as an example in this study), the absorption peaks at 1095 cm^−1^ and 1629 cm^−1^ in the 185-SCND curve are assigned to Si-O-Si stretching and N-H bending vibrations, respectively. The characteristic peaks of O-Si-O and Si-O-Si at 850 cm^−1^ and 1000–1200 cm^−1^ in the 185-SCND-KH560-M2070 curves indicate that KH560 acts as a linker to covalently bind KH560-M2070 to the SCND surface through dehydration condensation [25]. Additionally, a broad peak observed in the SCND sample at approximately 3482 cm^−1^, which disappears after reaction with KH560-M2070, is attributed to hydroxyl (-OH) groups on the surface of the diamond nanosheets. The reaction of these -OH groups with KH560-M2070 leads to the formation of covalent bonds, resulting in the disappearance of the broad peak.

XRD analysis was further used to evaluate the crystallinity and phase structure of the diamond nanosheets (Figure 2b). The XRD patterns showed that SCND samples with different sizes displayed distinct diffraction peaks at 2θ ≈ 44 °C and 2θ ≈ 75 °C for the diamond (111) and (200) crystal planes, indicating that the samples exhibited good crystallinity. Specifically, the wider full width at half maximum (FWHM) observed in the 70 and 120 nm single-layer carbonitride nanosheet (SCND) samples indicated smaller grain sizes, while the 185 nm sample exhibited sharper peaks indicative of larger grain sizes [26]. Notably, no characteristic peaks of the graphite phase were observed in the XRD pattern of all the samples, confirming the high-purity diamond structure of the samples. These XRD results emphasized the high degree of consistency and purity of the diamond nanosheets in terms of crystal structure, and indicated the influence of grain size on the XRD diffraction properties.

Raman spectroscopy (Figure 2c) was used to elucidate the structural properties of the 185 nm SCND and corresponding SCND-KH560-M2070 samples. The SCND flakes exhibited a typical diamond characteristic peak at 1325 cm^−1^ in the Raman spectra, while the chemically modified SCND-KH560-M2070 samples demonstrated a slight peak shift to 1328 cm^−1^; a change that could be attributed to changes in the stress or chemical environment caused by surface modification [27].

TGA curves were used to analyze the organic shell content of KH560-M2070 in SCND-KH560-M2070. As shown in Figure 2d, the weight of SCND-KH560-M2070 remained relatively stable until 300 °C, indicating that SCND was highly thermally stable. However, decomposition of the organic shells started to occur above this temperature until almost complete decomposition at about 400 °C, which was calculated as 11.05 wt% of the mass fraction of stable SCND in SCND-KH560-M2070.

The chemical state and elemental composition of SCND-KH560-M2070 were determined by XPS (Figure 2e,f). The specific binding energy peaks in the Si 2p and N 1s in the XPS spectra revealed a chemical modification process on the diamond surface, indicating the formation of chemical bonding between KH560 and M2070 on the diamond nanosheet surfaces [28].

**Figure 3:**
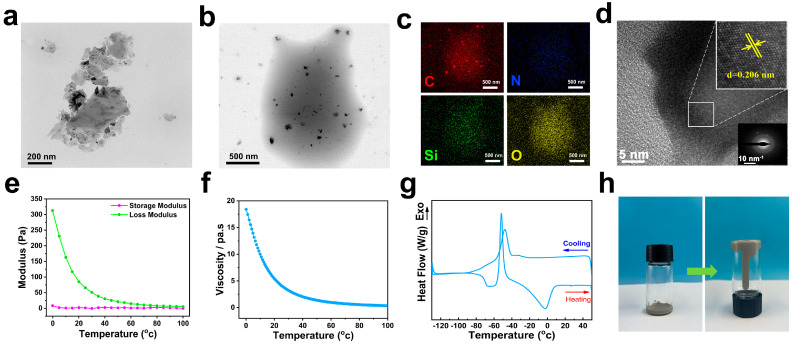
TEM images of SCND (**a**) and SCND-KH560-M2070 (**b**), EDS spectra of diamond nanosheets in SCND-KH560-M2070 at high magnification (**c**) and their lattice spacing and diffraction peaks (**d**), temperature-modulus curves (**e**), temperature-viscosity curves (**f**), DSC curves and (**g**), in-kind flow optics photographs of SCND-KH560-M2070 (**h**).

High-resolution transmission electron microscopy (HRTEM) observations of SCND and SCND-KH560-M2070 were carried out to assess the morphology and lattice structures of the samples. Figure 3a shows that SCDN is a sheet structure, and its SCND-KH560-M2070 (Figure 3b) obtained after graft modification shows that there is an obvious organic shell on the surface of SCND; and the appearance of this structure confirms that the present experiments have successfully introduced the organosilane KH560 and the polyether amine M2070 onto the surface of diamond nanosheets to form a stable organic–inorganic hybridized material. The EDS spectra (Figure 3c) even showed obvious enrichment of C, N, and Si elements. The spatial distribution of these elements was closely associated with the chemicals employed during the chemical modification procedure. Specifically, carbon was predominantly sourced from the diamond nanosheets, while silicon and nitrogen were introduced through the agents KH560 and M2070. This finding was further evidence of the success of the chemical modification, where the organic layer in this region was composed of flexible oligomers of KH560 and M2070. By analyzing the high-resolution HRTEM images (Figure 3d), the lattice structure of the diamond nanosheets was observed, and the diffraction rings corresponding to the (111), (220), and (311) lattice planes in the electron diffraction pattern [29] were clearly identified. The observed diffraction patterns not only validated the integrity of the SCND’s lattice structure, but also demonstrated its high crystallinity.

The rheological and DSC measurements revealed the physical behavior of SCND-KH560-M2070 at different temperatures, including changes in modulus, viscosity properties, and key physical parameters. Specifically, SCND-KH560-M2070 exhibited typical fluidic properties, where the loss modulus (G″) was consistently higher than the storage modulus (G′) (Figure 3e), emphasizing the fact that SCND-KH560-M2070 was dominated by good energy dissipation properties throughout the range of tested temperatures, resulting in a distinctly liquid behavior [30,31,32,33]. This was visually verified by the experimental observations of SCND-KH560-M2070 placed in an inverted vessel (Figure 3h), in which the slow flow of the fluid along the vessel wall clearly demonstrated its good fluidity [34]. In addition, a careful study of the viscosity variation of SCND-KH560-M2070 with temperature revealed a significant decrease with increasing temperature (Figure 3f), which further emphasized that the diamond nanosheets modified by KH560 and M2070 exhibited a more flexible and adaptive flow behavior under high temperature conditions [32,35,36,37]. This temperature-sensitive viscosity change reflected the thermal stability and tunable fluid properties of SCND-KH560-M2070. Differential scanning calorimetry (DSC) results further confirmed the liquid character of SCND-KH560-M2070 at room temperature and their enhanced flow properties under high ambient temperatures (Figure 3g), which strongly correlated to the rheological measurements.

### 3.2. Tribological Properties

**Figure 4:**
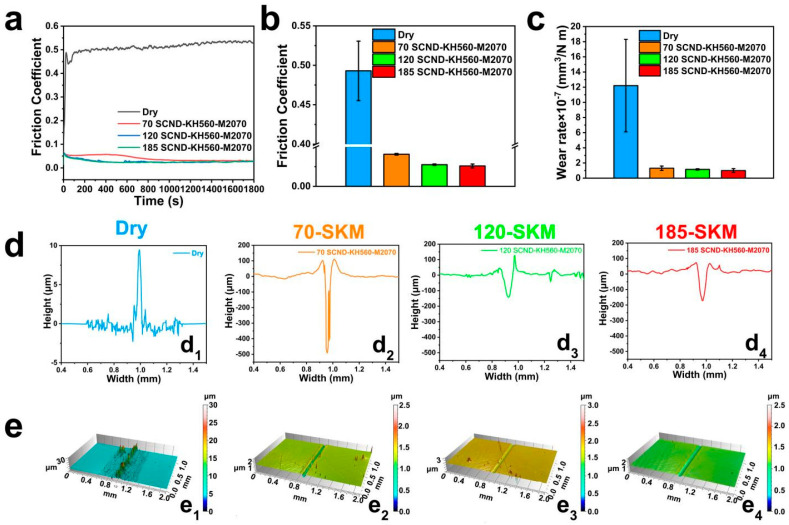
Friction coefficient graphs (**a**), friction coefficient histograms (**b**) and wear rate graphs (**c**) of the AISI52100 steel plates under different lubrication conditions, comparing the cross-sectional height contours (**d**) and their corresponding 3D contours (**e**) for dry and different SCND-KH560-M2070(SKM) lubrication sizes; where, in the lubrication condition representation, dry means dry friction, 70-skm means 70nm size SCND-KH560-M2070, and so on. In subfigures (**d_1_**–**d_4_**) and (**e_1_**–**e_4_**), the lubrication conditions are defined as follows: (**d_1_**,**e_1_**) under dry friction (no lubrication); (**d_2_**,**e_2_**) under lubrication with 70 nm SCND-KH560-M2070; (**d_3_**,**e_3_**) under lubrication with 120 nm SCND-KH560-M2070; and (**d_4_**,**e_4_**) under lubrication with 185 nm SCND-KH560-M2070.

The lubricating potential of SCND-KH560-M2070 as lubricants on macroscopic friction behavior was systematically evaluated. Through ball-to-plate friction tests using GCr15 steel balls with a surface roughness of 6 nm against an AISI 52100 steel plate with dimensions of 10 mm × 10 mm × 3 mm, the COF of the AISI 52100 steel substrate under ambient conditions was analyzed in detail. The experiments were carried out using a standard applied load of 5 N, and the friction properties of substrates lubricated with SCND-KH560-M2070 were compared. The experimental results (Figure 4a) revealed that the coefficient of friction between the steel ball and bare AISI 52100 steel plate under dry friction remained at a high level, at about 0.52. Significant friction reductions were observed with the introduction of the diamond nanofluids. Specifically, in the friction test using 70 nm diamond nanofluid lubrication, the COF of the AISI 52100 steel plate decreased to 0.054 during break-in after approximately 100 s, and further decreased to approximately 0.03 throughout the friction test period. The coefficient of friction was slightly lower than the 70 nm fluid when using the 120 nm diamond nanofluid, dropping to 0.027 after about 310 s of abrasion and then stabilizing at 0.029 at about 900 s. By contrast, for the 185 nm diamond nanofluid, the lowest COF value among all samples was observed, dropping to 0.025 at about 325 s of grinding and remaining low throughout the friction process (Figure 4a) [38]. The average steady-state COF values of the diamond nanoparticles with different sizes obtained from independent friction tests (Figure 4b) indicated that the average COF values of the 70, 120, and 185 nm ND nanofluids decreased by 91.7%, 94.3%, and 94.7% [39,40], respectively, compared to the unlubricated AISI 52100 steel substrate. According to the resulting data, the highest friction coefficient and worst lubrication were achieved under a dry friction condition, while the use of diamond nanofluids with different sizes significantly enhanced the lubrication performance. In particular, the larger sizes (185 nm) showed more significant performance in terms of lowering the coefficient of friction.

In addition, the friction region of diamond nanofluids with different sizes was characterized, and the average wear rate was calculated after multiple friction evaluations (Figure 4c) to reveal the differences in the friction performance of diamond nanofluids of different sizes. A three-dimensional white light interferometer technique was used, and representative contours as demonstrations in multiple tests (Figure 4d,e) to systematically evaluate the effectiveness of diamond nanofluids in reducing frictional wear. Specifically, under dry friction conditions without the application of diamond nanofluid lubrication, the abrasion trace trajectories appearing on the AISI 52100 steel substrate surface (Figure 4(e_1_)) revealed the intense adhesive wear experienced by the friction partner during the friction process. By analyzing the cross-section height profile (Figure 4(d_1_)), it was observed that the maximum height of the abrasion trace trajectory reached 8.98 μm, and this significant bulge indicated the phenomenon of metallic material transfer during the friction process. Specifically, the material on the surface of the steel ball adhered and built up on the surface of the steel plate under the action of friction [41]. By contrast, when diamond nanofluid was used as the lubricant, all nanofluid sizes significantly reduced the wear of the friction pair. Specifically, in friction tests that applied 70 nm diamond nanofluid lubrication, the depth of the abrasion marks on the AISI 52100 steel substrate was significantly reduced to only 498 μm (Figure 4(d_2_)), and the observed abrasion depths were further reduced to 173 μm with the use of the 120 nm fluids. Under lubrication conditions with 185 nm diamond nanofluids, the wear on the AISI 52100 steel substrate was the smallest, where the depth of the wear marks was only 152 μm (Figure 4(d_4_)). In addition, the wear volume data derived based on 3D white light interferometry indicated that diamond nanofluid lubrication sizes of 70, 120, and 185 nm decreased the wear volume of the AISI 52100 steel substrate by 89.3%, 90.6%, and 91.8% [42], respectively, compared to the unlubricated AISI 52100 steel substrate (Figure 4c). These findings highlighted the significant impact of dry friction action on the wear mechanism at the friction interfaces under lubricant-free conditions, and the potential benefits of diamond nanofluids in preventing direct metal-to-metal contact and reducing wear.

**Figure 5:**
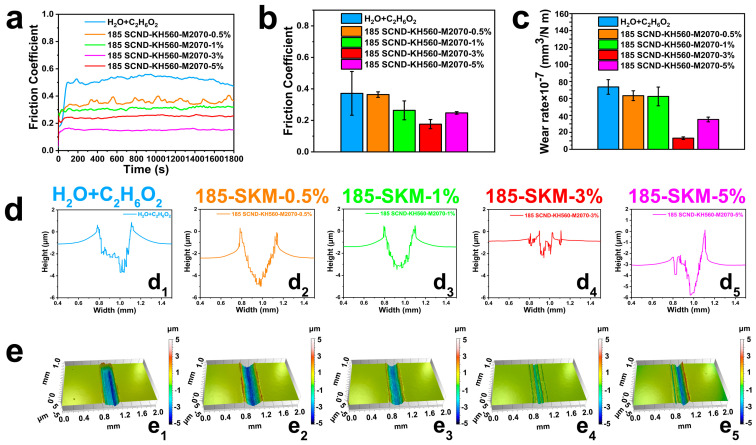
Friction coefficient graphs (**a**), friction coefficient histograms (**b**) and wear rate graphs (**c**) of the AISI 52100 steel plates under different lubrication conditions, as well as the cross-section height contours (**d**) and their corresponding three-dimensional contours (**e**) of the lubrication conditions with different contents of H_2_O-C_2_H_6_O_2_ and SCND-KH560-M2070. In the lubrication condition representation, H_2_O-C_2_H_6_O_2_ consisted of a 1:1 mixture of water and ethylene glycol; 185-SKM-0.5% denotes SKM obtained from diamond nanosheets with a size of 185 nm, which was added to aqueous ethylene glycol as a lubricant additive at a content of 0.5 wt%, and so on. In subfigures (**d_1_**–**d_5_**) and (**e_1_**–**e_5_**), the lubrication conditions are defined as follows: (**d_1_**,**e_1_**) under pure H_2_O-C_2_H_6_O_2_ lubrication; (**d_2_**,**e_2_**) under lubrication with 0.5 wt% 185-SKM; (**d_3_**,**e_3_**) under lubrication with 1 wt% 185-SKM; (**d_4_**,**e_4_**) under lubrication with 3 wt% 185-SKM; and (**d_5_**,**e_5_**) under lubrication with 5 wt% 185-SKM.

The performance of diamond nanofluids as additives for water–glycol lubricants was investigated, based on the 185-size diamond nanofluid—which has the best performance at different sizes—and the effect of its different contents on the lubricating behavior of the lubricant. The results showed that mixtures of H_2_O-C_2_H_6_O_2_ with different concentrations of SCND-KH560-M2070 exhibited significant differences in COF reduction and wear reduction (detailed data provided in Figure 5). In the case of H_2_O-C_2_H_6_O_2_, as shown in Figure 5a, the COF of H_2_O-C_2_H_6_O_2_ stabilized at about 0.46 after a 150-s break-in period, resulting in a relatively high average COF of about 0.372 (Figure 5b) [43]. When H_2_O-C_2_H_6_O_2_ was added with 0.5 wt% SCND-KH560-M2070, the COF was decreased to approximately 0.35; a reduction of only 25.2%. Weak friction reduction and wear resistance were observed because the low concentration of SCND-KH560-M2070 could not form a continuous and stable protective film between the friction sub-surfaces. As the concentration of SCND-KH560-M2070 increased, a 1 wt% addition significantly enhanced lubrication performance and provided stable COF during sliding. The improvement in friction and wear performance was evident when the COF value dropped to about 0.31 after about 75 s of break-in and continued to steadily fluctuate, compared to pure aqueous glycol, where the COF dropped to about 0.31. Thus, H_2_O-C_2_H_6_O_2_ with 1 wt% SCND-KH560-M2070 exhibited a COF reduction of 34.8%, with a corresponding wear volume loss of about 26.5%. When the concentration of SCND-KH560-M2070 increased to 3 wt%, a stable friction profile and corresponding lowest friction coefficient and wear volume were observed. The COF dropped to 0.16 at about 82 s of break-in and remained low throughout the friction process. Compared to H_2_O-C_2_H_6_O_2_, H_2_O-C_2_H_6_O_2_ with 3 wt% SCND-KH560-M2070 showed a 66.9% reduction in COF, with a corresponding wear volume loss of about 81.8%, resulting in optimal friction reduction and anti-wear properties [44]. This significant improvement in lubrication properties was attributed to the homogeneous dispersion and shear slip of SCND-KH560-M2070 in H_2_O-C_2_H_6_O_2_. In addition, the high surface area of the nanodiamond flakes combined with the polar portion of KH560-M2070 contributed to the adsorption of SCND-KH560-M2070 on the steel surface, forming a protective film that further prevented severe wear in the friction pairs. When the additive content was 5 wt%, the COF of 5 wt%185-SCND-KH560-M2070 continued to steadily fluctuate after the COF decreased to 0.25 during the friction test after about 45 s of wear, and aqueous ethylene glycol containing 5 wt% of the SCND-KH560-M2070 had a COF reduction of 46.1%, with a corresponding loss of wear volume of about 55.1%, compared to H_2_O-C_2_H_6_O_2_. In this study, all four lubricant combinations exhibited better stability of COF than pure aqueous glycol, which could be attributed to the formation of the diamond nanofluid friction film, with 3 wt% exhibiting the lowest coefficient of friction.

To more visually analyze the differences in wear rates induced by changes in the concentration of diamond nanofluids (SCND-KH560-M2070) within the H_2_O-C_2_H_6_O_2_ solvent system, a detailed analysis of the wear regions was performed. By analyzing the cross-section height profile (Figure 5d) and 3D profile (Figure 5e), it was observed that the geometric characteristics of the abrasion marks and abrasion widths obtained in pure aqueous ethylene glycol solvents and in the system containing 0.5 wt% SCND-KH560-M2070 presented similarities. The maximum height of the H_2_O-C_2_H_6_O_2_ abrasion tracks reached 5.14 μm (Figure 5(d_1_)). However, as the concentration of SCND-KH560-M2070 increased to 1 wt%, a significant decrease in the width of the wear marks was observed, which characterized the enhancement of the lubrication effect, and the depth of the wear marks was significantly reduced to only 3.52 μm for the AISI 52100 steel substrate (Figure 5(d_3_)). Significantly, as the concentration of SCND-KH560-M2070 increased to 3 wt%, the width of the abrasion marks was minimized, with the most minimal wear on the AISI 52100 steel substrate, with abrasion depths of only 2.31 μm (Figure 5(d_4_)). This implied that the anti-wear performance was optimized at this concentration. Conversely, a further increase of SCND-KH560-M2070 to 5 wt% resulted in an inverse increase in abrasion mark width and an increase in observed abrasion mark depth to 5.87 μm. This possibly reflected the inverse effect of excessive diamond nanofluid concentration in the lubrication system. In addition, wear volume data derived, based on three-dimensional white light interferometry, showed that the diamond nanofluid lubrication systems with 1 wt%, 3 wt%, and 5 wt% sizes reduced the wear volume of the AISI 52100 steel substrate by 26.5%, 81.8%, and 55.1%, respectively, compared to H_2_O-C_2_H_6_O_2_ (Figure 5c). These results confirmed the significant advantage of different contents of diamond nanofluids in improving the anti-wear performance of friction interfaces when the concentration of SCND-KH560-M2070 increased to 3 wt%.

### 3.3. Lubrication Mechanism

**Figure 6:**
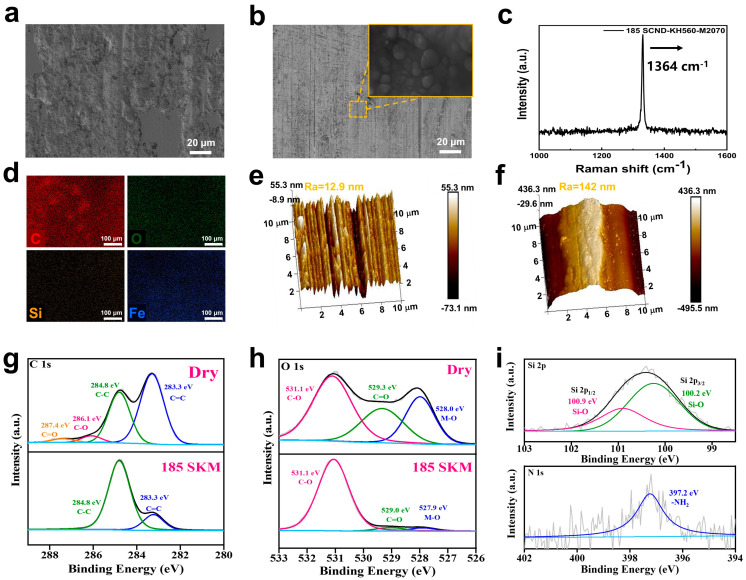
Wear surfaces of the AISI 52100 steel plate under dry friction conditions (**a**) and diamond nanofluid lubrication conditions (**b**), Raman spectroscopy of the wear surface under diamond nanofluid lubrication (**c**), elemental analysis under lubricated conditions (**d**), surface roughness examined by AFM under dry friction (**f**) and lubricated conditions (**e**), and surface chemistry analyzed by XPS under dry friction (**g**,**h**) and lubricated conditions (**i**).

To better investigate the anti-wear performance of SCND-KH560-M2070, the morphology of the wear surface was characterized in detail. As shown in Figure 6a, wear was extremely serious in the AISI 52100 steel plate under dry friction conditions, because the lubrication failure produced a wide and deep wear track, and its surface morphology also changed significantly, demonstrating a concave-convex state; and obvious micro-cracks accompanied by grooves were observed, possibly due to dry friction resulting in serious adhesive wear [45]. However, the surface of the AISI 52100 steel plate after lubrication with the diamond nanofluid was flat and almost invisible to wear (Figure 6b) [46], which was attributed to the good dispersion of diamond nanofluid in the steel matrix; and this prevented cracks from expanding in the steel matrix [47]. A substance similar to the diamond morphology was observed in the surfaces of the scratches in the high-magnification image (Figure 6b). Figure 6c shows the Raman spectra of the wear surface of the AISI 52100 steel plate under diamond nanofluid lubrication conditions. A characteristic Raman peak was clearly identified in the spectrum, located at 1364 cm^−1^, which corresponded to the characteristic absorption peak of the diamond nanosheets. Combined with EDS (Figure 6d) analysis, this phenomenon confirms that diamond nanofluids form a diamond protective film on the steel plate surface during friction. The formation of this protective film effectively realized low friction and low wear. Meanwhile, combining the AFM images of the two steel plate surfaces (Figure 6e,f), the surface of the AISI 52100 steel plate in the diamond nanofluid lubrication state was smoother, with an average surface roughness of Ra = 142 nm, while the surface of the AISI 52100 steel plate in the dry friction state was rougher, with an average surface roughness of Ra = 12.9 nm. These results were also consistent with the analysis of the SEM images mentioned above [48].

Detailed XPS analyses (Figure 6g–i) provided insights into the chemical effects of diamond nanofluids (SCND-KH560-M2070) on the wear surface of the AISI 52100 steel plates. Under dry friction conditions, the C1s spectra of the steel plate surface revealed the presence of multiple chemical bonds, as reflected in the C=O, C-O, C-C, and C=C structures, and these signal peaks were located at 287.4, 286.1, 284.8, and 283.3 eV, respectively. At the same time, the peaks in the O1s spectra were at 531.1, 529.3, and 528.0 eV; and these characteristic peaks strongly suggested that the steel surface underwent oxidation [49].

By contrast, the surface of the steel plate after coating with the diamond nanofluid significantly exhibited signal enhancements of the Si and N elements, as indicated by the 100.9 and 100.2 eV of the Si 2p signal peaks, and the 397.2 eV of the N 1s signal peak [50]. This suggested that the silicon and nitrogen components in the diamond nanofluid were transferred to the steel plate surface during mechanical friction, forming a uniform and continuous lubricating film. This lubricating film not only provided physical separation of the friction interface, reducing direct metal-to-metal contact, but also resulted in chemical protection that helped to stabilize the chemical state of the steel plate surface, reducing the potential for oxidation.

**Figure 7:**
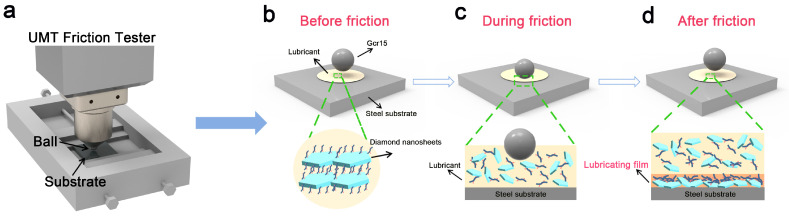
Schematic representation of the UMT friction testing device (**a**) and the lubrication mechanism of solvent-free diamond nanofluid: (**b**) pre-friction, (**c**) during friction, and (**d**) post-friction.

Based on the experimental data, the lubrication mechanism of the diamond nanofluids (Figure 7) was analyzed. The functionalized polymer layers introduced on the surface of the diamond nanosheets formed a flexible protective shell that mitigated mechanical damage to the diamond sheet layer. During friction, these nanosheets formed a hard anti-wear layer, while the polymer layers provided flexibility, and the two worked together to form a strong and elastic anti-wear film on the contact surface.

At the onset of friction, the functionalized polymer layers on the diamond nanosheets enhanced their uniform adhesion to the substrate due to their high affinity for the contact surface. As the stress increased, the spheres on the friction surface began to interact with the nanofluid, and a stable wear-resistant film rapidly formed through multilayer adsorption of the functionalized polymer layers; a process that had no significant break-in period. The good dispersion of the diamond nanofluids allowed the material to continuously enter the contact zone and constantly replenish the adsorbed layer, promoting the formation of a protective wear film. The presence of the flexible polymer layers not only enhanced the stability and durability of the wear film, but also helped maintain a stable coefficient of friction [19,22,51].

During continuous testing, the nanofluid progressively covered the friction surface, forming a wear-resistant film similar to the substrate surface, which promoted excellent lubrication. Through this mechanism, the functionalized polymer layers on the diamond nanosheets contributed to the formation of a protective wear film, exhibiting excellent performance in the lubrication process.

## 4. Conclusions

In this experiment, a solvent-free diamond nanofluid, SCND-KH560-M2070, was prepared by covalently grafting KH560 with M2070 to form a KH560-M2070 intermediate, which was subsequently grafted onto SCND, and its frictional properties were investigated as a lubricant as well as a lubricant additive in an aqueous glycol system, respectively.

When diamond nanofluids were used as lubricants, the 185 nm diamond nanofluids exhibited excellent macroscopic lubrication properties. Compared to the dry friction state, the nanofluid could effectively reduce the COF to 0.025—a reduction of 94.7%—and the wear rate was also reduced by 96%. Further experiments were conducted to evaluate the lubrication effect of 185 nm diamond nanofluids as additives in aqueous ethylene glycol systems at different concentrations. The experimental results showed that the diamond nanofluids decreased the COF to 0.16 at a concentration of 3 wt%, which was 66.9% lower than the aqueous ethylene glycol solution; while the wear rate was reduced by 81.8%.

The lubricating effect of diamond nanofluids mainly originated from the functionalized polymer layers on their surfaces. These layers provided protection for the diamond flake layer and contributed to the rapid formation of a strong and elastic wear film, significantly reducing the coefficient of friction (COF) and improving wear resistance, demonstrating its potential as an advanced lubricant material.

However, some limitations of this research should be noted. For instance, the experimental conditions were limited to specific load and frequency ranges, which may not fully represent real-world applications. Additionally, the study focused only on specific sizes and concentrations of diamond nanosheets, and the long-term performance of the nanofluids under extreme conditions, such as high temperatures or prolonged use, was not investigated. The underlying lubrication mechanisms at the molecular level also require further exploration.

Based on the findings of this study, future research could focus on the following areas: investigating the effects of different surface modifiers on the lubrication performance of diamond nanosheets; expanding the experimental conditions to include extreme environments such as high temperatures and heavy loads; utilizing molecular dynamics simulations or in situ characterization techniques to further understand the lubrication mechanisms; optimizing the preparation processes of diamond nanofluids to improve their stability and applicability in industrial lubrication.

## Figures and Tables

**Table 1:** Table of fixed parameters for friction testing conditions.

Parameter	Value	Unit	Description
Load	5	N	Constant load applied in the friction test
Frequency	5	Hz	Frequency of the friction test
Test duration	30	min	Duration of each friction test
Steel plate size	2 × 2 × 1	cm^3^	Size of AISI 52100 steel plate
Steel ball diameter	6	mm	Diameter of GCr15 steel ball
Steel ball material	GCr15		Material of the steel ball used in the friction test
Steel plate material	AISI 52100		Material of the steel plate used in the friction test
Steel ball surface roughness	6	nm	Surface roughness of GCr15 steel ball
Steel plate surface roughness	12.9	nm	Surface roughness of AISI 52100 steel plate

**Table 2:** Table of adjustable parameters in friction experiments.

Condition	Parameter	Value	Unit	Description
Nanofluid size	Diamond nanosheet size	70, 120, 185	nm	Three different sizes of diamond nanosheets
Nanofluid concentration	Nanofluid concentration	0.5%, 1%, 3%, 5%	wt%	Different concentrations of diamond nanofluids

## Data Availability

The original contributions presented in this study are included in the article. Further inquiries can be directed to the corresponding authors.
